# Lipid Indexes and Quality Evaluation of Omega-3 Rich Oil from the Waste of Japanese Spanish Mackerel Extracted by Supercritical CO_2_

**DOI:** 10.3390/md20010070

**Published:** 2022-01-13

**Authors:** Vikash Chandra Roy, Jin-Seok Park, Truc Cong Ho, Byung-Soo Chun

**Affiliations:** 1 Department of Food Science and Technology, Pukyong National University, 45 Yongso-ro, Nam-gu, Busan 48513, Korea; vikashft@hstu.ac.bd (V.C.R.); jin1931@pukyong.ac.kr (J.-S.P.); hocongtruc@mku.edu.vn (T.C.H.); 2 Department of Fisheries Technology, Hajee Mohammad Danesh Science and Technology University, Dinajpur 5200, Bangladesh; 3 PL MICROMED Co., Ltd., 1F, 15-5, Yangju 3-gil, Yangsan-si 50620, Gyeongsangnam-do, Korea

**Keywords:** supercritical-CO_2_, marine oils, omega-3 fatty acids, lipid index, fishery by-products

## Abstract

Japanese Spanish mackerel (JSM) (*Scomberomorus niphonius*) is a marine fish species containing health-beneficial polyunsaturated fatty acids (PUFAs). In the present study, the quality of JSM by-products oils extracted by supercritical CO_2_ (SC-CO_2_) and organic solvent extraction was compared in terms of physico-chemical properties of the oils. Eicosapentaenoic acid (EPA) is one of the important polyunsaturated fatty acids present in SC-CO_2_-extracted skin and muscle oil 5.81 ± 0.69% and 4.93 ± 0.06%, respectively. The amount of docosahexaenoic acid (DHA) in SC-CO_2_-extracted skin and muscle oil was 12.56 ± 0.38% and 15.01 ± 0.28%, respectively. EPA and DHA are considered as important PUFAs for the development of brain function and the prevention of coronary heart diseases. Extracted oils showed considerable antioxidant activity. In the obtained oils, atherogenic index (AI) values varied from 0.72 to 0.93 and thrombogenic index (TI) ranged from 0.75 to 0.92, which is considered an acceptable level. Fatty acid composition, bio potentiality, thermogravimetric, and vitamin D analysis showed that oils extracted from JSM by-products can be a good source of oil for application in food, pharmaceutical and cosmetic industries. Therefore, the present research revealed the potentiality of green valorisation of *S. niphonius* by-products as a possible sustainable approach for targeting the era of zero waste.

## 1. Introduction

Every year, tons of fishery wastes are produced from fishery processing [1,2]. The utilization of these wastes can save our environment from pollution; moreover, adequate processing of these wastes can be an important source of different valuable compounds, such as fish oil [3,4,5]. However, these by-products have been discarded in the environment, utilized as feed ingredients for farmed organisms or used as organic fertilizers in agricultural fields. Practically, there is a huge possibility for using this material as a good source of human food grade dietary oil polyunsaturated fatty acids (PUFA) supplements. Recently, the demand for fish oil containing long-chain omega-3 fatty acids has increased due to their health beneficial effectiveness against cardiovascular diseases [6], development of neural systems and in boosting memory power, etc. [2,3,4,7,8]. Eicosapentaenoic acid (EPA) and docosahexaenoic acid (DHA) are PUFA that are available in marine fish oils [2]. Previous reports also showed that fish oil is also a good source of different kinds of fat-soluble vitamins, such as vitamin A, D, E, K and co-enzyme 10 (Q_10_) [4].

Mackerel is a well-known epipelagic fish species containing a high amount of oil in its body [5,9,10,11] and a high amount of unsaturated fatty acids, including a higher percentage of PUFA, which are very helpful for human health. Japanese Spanish mackerel (*Scomberomorus niphonius*) (JSM) is mostly distributed in the temperate waters of Korea, Japan, and China [12]. JSM (*S. niphonius*) is an important fish species in Asia, and very popular for sushi, as well as grilled and pan-fried food [13]. Processing by-products, skin, bone, head, blood, and viscera represent 42.2–44.5% of its total weight [14]. These by-products can be used for providing a good source of edible lipid for meeting the increasing demand for PUFA-containing oil throughout the world [1]. De-oiled residues can be used as a high-protein concentrate source for farmed organisms such as cattle, poultry, and fish feed [15].

Conventional oil extraction methods using *n*-hexane or ethanol, or other different organic solvents require a further purification step to remove the solvents. This process is time-consuming. Thermal treatment during the extraction and separation steps might also have some negative effects on the stability of its unsaturated fatty acids, which have a higher tendency to oxidise [16].

Supercritical carbon dioxide extraction (SC-CO_2_) is a new and promising technology for the extraction of oils from raw materials [17]. SC-CO_2_ extraction is an environmentally friendly, non-toxic, inexpensive process, and does not need any further purification of extracted oils [18]. CO_2_ is cheap and commercially available throughout the world. The solubility power of SC-CO_2_ can be controlled by controlling the pressure and temperature [16].

Thousands of tons of JSM are produced commercially throughout the world [19]; however, there are very few studies on the extraction of oil from different parts of the fish by SC-CO_2_ compared with the conventional extraction. The primary objectives of this study are to extract the oil from different parts of JSM, both by SC-CO_2_ and Soxhlet (*n*-hexane) extraction, to evaluate the physical properties, oil stability, radical scavenging activities (2, 2′-azino-bis-3-ethylbenzothiazoline-6-sulfonic acid (ABTS^+^), and 1,1-diphenyl-2-picrylhydrazine (DPPH)), to compare fatty acids, analyse the lipid indexes, thermal quality, and availability of vitamin D to determine the suitability of the extracted oils for the further utilization of different value-added products.

## 2. Results and Discussion

### 2.1. Characterization of the Wastes

Separated JSMs were divided into the skin, muscle, bone, head, and viscera, and the remaining blood and slimes were divided into other portions. More than half of the body parts were the remaining edible muscle (50.89 ± 0.30%), whereas the waste percentages were nearly 50%. Among them were skin, which contributed 8.61 ± 0.34%, and bone, head, and viscera, which, respectively contributed 7.91 ± 0.14%, 16.03 ± 0.17%, and 6.22 ± 0.22% (Table S1, Supplementary Materials). Leu et al. [14] reported that 53.5 ± 4.3% edible muscle can be obtained from Atlantic mackerel and the remaining portion is considered as a by-product, in which skin, bone, head, and visceral portions contributed 10.3 ± 1.3%, 6.6 ± 1.2%, 17.1 ± 0.8%, and 10.9 ± 3.7%, which is almost similar to our experimental fish species.

After freeze-drying, the raw materials proximate compositions were analysed to obtain the percentages of moisture, proteins, lipids, and minerals (Table S1, Supplementary Material). The highest percentage of oil was obtained from the skin 51.08 ± 0.01%, whereas muscle, bone, head, and viscera showed, 30.24 ± 0.47%, 42.50 ± 1.03%, 42.27 ± 0.96%, and 30.27 ± 1.08%, respectively (Table S1, Supplementary Materials).

### 2.2. Comparison of the Yield

**Figure 1:**
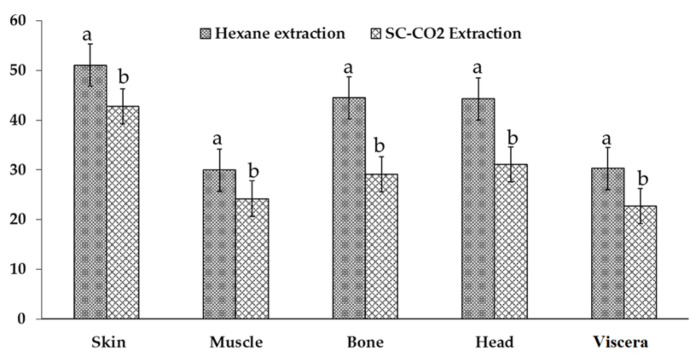
Comparison of the yield of supercritical carbon dioxide and *n*-hexane extractions (*n* = 3). Different superscripts in the same group (skin, muscle, bone, head, and viscera) indicate the significant differences (*p* < 0.05).

Amount of extracted oil from the dried raw materials is affected by the extraction procedures. The highest yield of oil was obtained from the Soxhlet extraction of skin (51.08 ± 1.35%) (Figure 1), followed by bone, head, viscera, and muscle (44.51 ± 1.13%, 44.27 ± 1.81%, 30.27 ± 1.15%, and 29.94 ± 0.95%). SC-CO_2_ shows a slightly lower yield with 42.79 ± 1.79%, 24.18 ± 1.09%, 29.11 ± 1.81%, 31.08 ± 2.01%, and 22.70 ± 1.35% from the skin, muscle, bone, head, and viscera, respectively, on a dry weight basis. The recovery percentage of the oils was highest in the skin (83.77%) in SC-CO_2_ extraction; however, the bone showed a lower recovery rate (65.41%). The yield of the solvent extraction was high due to the efficiency and poor selectivity of the *n*-hexane for extracting all classes of lipids. Haq et al. [16] reported that nearly 86.99% of oil can be recovered from the by-products of salmon at 45 °C and 25 MPa compared to solvent extraction. Sahena et al. [9] also reported that 53.6% of the oil was recovered from the skin of the Indian mackerel. SC-CO_2_ extraction is safe due to the non-toxicity of the solvents. Besides, CO_2_ can easily separate from the extracted oil and leaves no traces in the final oil [20,21].

### 2.3. Colour and Viscosity

Colour is one of the important parameters of oil products. The colour properties of the oils extracted from different body parts using Soxhlet and SC-CO_2_ extraction were analysed using instrumental colour measure L*, a*, and b* systems (Table 1). The SC-CO_2_-extracted head oil had the highest lightness index (L*) (32.06 ± 0.12), whereas the highest a* value was obtained from the Soxhlet-extracted viscera oil (9.65 ± 0.38) and the highest b* value was obtained in bone oil from the same extraction. The colour is an important factor for increasing acceptability, as further costs are required to obtain acceptable light-coloured oil [22].

The viscosity of the oils from both extractions showed a significant difference. SC-CO_2_-extracted oils showed lower viscosity compared to the Soxhlet-extracted oils (Table 1). The lower value of the SC-CO_2_-extracted oils indicates that it has high purity and contains higher smount of unsaturated fatty acids [23] in comparison with the Soxhlet extracted oils [16]. Previous studies reported that the possible reason for increasing the viscosity is the impurity in the crude oil [16,18,24].

### 2.4. Oxidative Quality of the Oils

Three important parameters, acid value (AV), peroxide value (PV), and free fatty acid value (FFA), were analysed to determine the quality of the extracted oils (Table 2). Hexane-extracted oils showed comparatively higher AV than SC-CO_2_-extracted oils. The highest AV was found in hexane-extracted fish muscle oil (10.84 ± 0.25 mg KOH/g of oil), whereas SC-CO_2_-extracted muscle oil showed lower AV (7.13 ± 0.08 mg KOH/g of oil). AV is an indicator of FFA and other non-lipid acid compounds such as acetic acids, mainly formed by the hydrolysis reaction of triglycerides during the spoilage of raw materials [25]. Exposure of atmospheric oxygen to hot moist air is one of the possible causes for hydrolysis of the oil [16]. All the oils extracted by SC-CO_2_ extraction showed the values within the acceptable limit (7–8 mg KOH/g of oil), whereas *n*-hexane-extracted oils showed beyond the acceptable limit (>9). Researchers have suggested that there are several factors, such as the composition of the oil, extraction techniques, and the freshness of the raw materials, regarding the quality of fish oil [3,9,16,18,26].

PV determines the quantification of the hydro-peroxides present in the crude oil due to the oxidation process [27]. Compounds such as FFA, aldehydes, and alcohols are formed due to further breakdown of the oxidized products [3]. Oils extracted in both methods showed PV ranging from 1.06 ± 0.04 to 1.65 ± 0.06 meq/kg, which were within the acceptable limit (≤5 meq/kg) [5,16,27]. Fish oil has a high amount of PUFA that is highly sensitive to oxidative damage. This is why methods that involve reduced contact with oxygen and temperature are preferred to extract the fish oil [16,27].

SC-CO_2_-extracted oils showed the lower FFA compared with *n*-hexane-extracted oils, although all the oils showed FFA values within the acceptable limit (1–7%) [16]. Sahena et al. [10] reported that Soxhlet-extracted tuna oils have higher FFA values than SC-CO_2_-extracted oils. Oils exposed to a high temperature for a long time comparatively have higher FFA values [28]. That is why SC-CO_2_-extracted oils showed better quality than *n*-hexane-extracted oils.

### 2.5. Antioxidant Activities

#### 2.5.1. ABTS^+^ Radical Scavenging Activity

**Figure 2:**
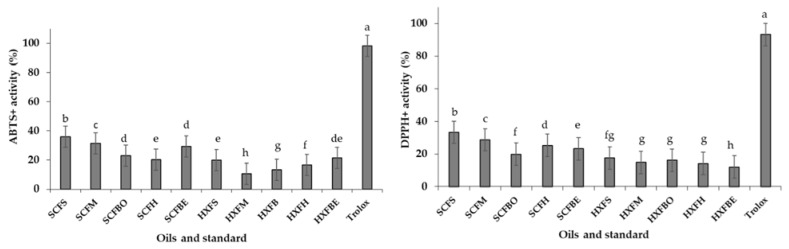
In vitro antioxidant activity of the extracted oils from different parts of JSM fish. *SCFS*, *SCFM*, *SCFBO*, *SCFH*, *SCFBE* stand for SC-CO_2_ extracted fish skin, muscle, bone head, viscera oils; *HXFS*, *HXFM*, *HXFBO*, *HXFH*, *HXFBE* stand for *n*-hexane extracted fish skin, muscle, bone, head, viscera; Different superscripts indicate the significant differences (*p* < 0.05).

Oils extracted from different body parts of the JSM using SC-CO_2_ and Soxhlet extractions showed a significant difference in terms of ABTS^+^ (azino-di, 3-ethylbenzthiazoline-6-sulfonic acid) radical scavenging activity (Figure 2). SC-CO_2_-extracted fish skin oil showed the highest ABTS radicals scavenging activity (36.03 ± 1.35%) just after the standard (Trolox 98.13 ± 1.82%). Haq et al. [16] reported that SC-CO_2_-extracted salmon skin oil showed 82.78% ABTS^+^ radical scavenging activity. SC-CO_2_-extracted oils showed higher ABTS^+^ activities compared to *n*-hexane-extracted oils. Low-ABTS^+^ radical scavenging activity was obtained from the *n*-hexane-extracted fish head oil.

#### 2.5.2. DPPH Radical Scavenging Activity

Higher DPPH (2,2-diphenyl-1-picrylhydrazyl) radical scavenging activity was obtained from the oil extracted from JM skin (33.35 ± 1.81%) in SC-CO_2_ extraction methods, whereas hexane-extracted viscera showed the lowest DPPH radical scavenging activity (12.00 ± 0.96%) (Figure 2). The obtained DPPH antioxidant results from the different body parts in both extraction techniques showed a significant difference. The variation of the antioxidant activity of the extracted oils could be dependent on the presence of the omega-3 fatty acids and quality indexes of the oils [6,29].

### 2.6. Fatty Acid Composition of the Oils

The fatty acid compositions of the oils extracted by Soxhlet, and SC-CO_2_ extraction methods were analysed. Table 3 represents 26 types of fatty acids from 37 types of fatty acids by authentic standards. SC-CO_2_-extracted fish oils from the skin, muscle, bone, head, and viscera showed 35.72%, 35.07%, 39.26%, 38.93%, and 39.84% total saturated fatty acids (SFA), respectively; however, in the Soxhlet extraction, the overall percentages of SFA increased considerably, having 38.28%, 36.18% 40.98%, 33.92%, and 37.02% in the above-mentioned parts.

Soxhlet-extracted by-product oils also showed increasing trends of monounsaturated fatty acids (MUFA). Table 3 shows that Soxhlet-extracted bone oils contain the highest amount of MUFA (44.60%) compared with other oils, whereas viscera oils contain less MUFA than other samples. Zuta et al. [30] reported 39.2%, 37.9%, and 43.8% MUFA were obtained from the Indian mackerel skin, muscle, and viscera, respectively.

**Figure 3:**
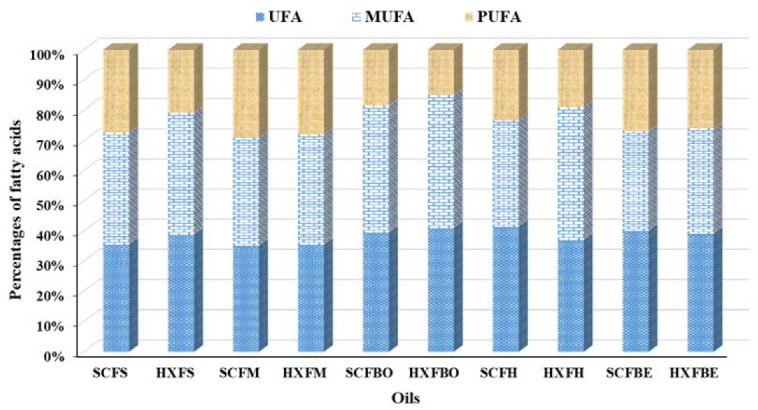
Comparison among the percentages of the fatty acids of the oils extracted from different parts of JSM fish. (*SCFS*, *SCFM*, *SCFBO*, *SCFH*, *SCFBE* stand for SC-CO_2_ extracted fish skin, muscle, bone head, viscera oils; *HXFS*, *HXFM*, *HXFBO*, *HXFH*, *HXFBE* stand for *n*-hexane extracted fish skin, muscle, bone, head, viscera oils; UFA stand for unsaturated fatty acids; MUFA stand for monounsaturated fatty acids; and PUFA stand for polyunsaturated fatty acids).

Table 3 shows that the two important PUFA, EPA and DHA, contributed the highest percentages in SC-CO_2_-extracted skin and muscle oils, corresponding to 5.81 ± 0.69%, 12.56 ± 0.38% and 4.93 ± 0.06%, 15.01 ± 0.28%. These PUFAs are considered for the development of the brain and the prevention of coronary heart diseases [8,31]. The total percentages of PUFA vary between 29.15% to 14.99% in SC-CO_2_-extracted muscle oil and Soxhlet-extracted bone oil. The overall percentages of PUFA decreased in Soxhlet extraction (Figure 3) as a result of its exposure to heat and atmospheric pressure during the extraction process [16]. Sahena et al. [9] reported that the highest PUFA was obtained from the skin, followed by muscle, viscera, and heads of Indian mackerel. Zuta et al. [30] also showed that PUFA from the skin, muscle, and visceral parts is, respectively, 22.6%, 21.2%, and 15.2% in Soxhlet-extracted Indian mackerel oil. The fatty acids profile of the fish oil depends on feeding habits and environmental conditions [32]. Thus, marine fish are distinguished by their higher concentration of EPA and DHA, since they are largely dependent on marine planktons [33].

### 2.7. Lipid Quality of the Extracted Oils

The ratios between omega-3(ω-3)/omega-6(ω-6), AI, and TI are presented in Table 3. The ω-3/ω-6 ratio is generally used for the analysis of lipid quality [34], and it varies from 1.25 to 3.55 in all of the oils extracted from JSM. Different studies reported that a high ω-3/ω-6 ratio is likely to be associated with different physiological problems such as cardiovascular diseases and cancer [35]. However, all the oils showed a very good ω-6/ω-3 ratio recommended by (ω-6/ω-3 = 10) FAO. AI values vary from 0.72 to 0.93 and TI ranges 0.75 to 0.92 in all the oils, whereas the acceptable level in fish oils varies between 0.33–1.47 (AI) and 0.16–1.15 (TI) depending on the species [36].

### 2.8. Thermal Property of the Oil

**Figure 4:**
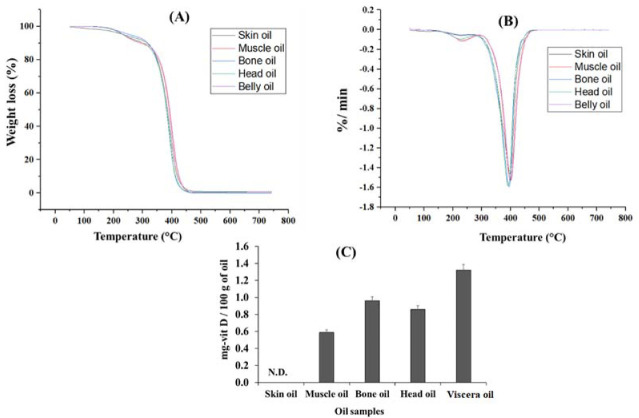
TGA (**A**), DTG (**B**), and Vitamin D analysis (**C**) of the oil extracted using SC-CO_2_ from different parts of Japanese Spanish mackerel.

Thermogravimetric (TGA) and differential thermogravimetric analysis (DTG) graphs of the SC-CO_2_ extracted by-products oils from JSM fish oils are presented in Figure 4. The obtained TGA properties showed an almost similar trend in thermal degradation at extrapolated onset temperature near 290 °C (Figure 4A). However, with close observation, muscle oil and viscera oil showed higher thermal stability compared to skin, head and bone oil. The possible reason for this might be the presence of comparatively higher saturated fatty acids present in muscle (39.26%) and viscera oil (39.84%) compared to skin (35.72%), head (38.39%), and bone (35.07%). Previous studies reported that the extrapolated onset temperature of fish oil was nearly at 270 °C [5]. Getachew et al. [18] reported that the stable lipid materials show lower weight loss during thermal degradation. The final degradation of all oils was 740 °C which indicates that SC-CO_2_ extracted oils from JSM by-products completely degraded when the temperature was up to 740 °C. DTG curves of the by-product’s oils are shown in Figure 4B. The degradation curve of the oil was found nearly at 405–430 °C, indicating that the obtained oils are suitable for application in high temperatures and are suitable for utilization in food, cosmetic, and pharmaceutical industries [5].

### 2.9. Analysis of Vitamin D

Vitamin D is often known as cholecalciferol (D_3_) in animal bodies, an important micronutrient that has a significant role in calcium homeostasis [37]. Although vitamin D is synthesized by human skin with the aid of sunlight, the modern lifestyle offers limited opportunity for sun exposure. The lack of vitamin D leads to various diseases such as osteoporosis, rickets, etc. [38,39]. Therefore, the demand and concern for vitamin D is increasing for those who are health-conscious. The presence of vitamin D in JSM oil is presented in Figure 4C. The different body parts of JSM oils contain vitamin D from 0.59 ± 0.01 mg/100 g oil to 1.32 ± 0.3 mg/100 g oil, indicating that these oils have a great potentiality for application in bio-functional foods.

## 3. Materials and Methods

### 3.1. Sample Preparation

JSM was bought from a local market in Busan, Republic of Korea, and transported to the laboratory in an ice box. The average sizes of the fish were 1.5 ± 0.28 kg. They were peeled manually, and the skin, muscle, bone, head, and visceral portions were separated. The separated portions were then washed thoroughly using chilled water, and freeze-dried using Hypercool HC4110 (Daejeon, Korea). The dried samples were ground using an electric blender. The crushed samples were stored at −24 °C until the oil was extracted.

### 3.2. Reagents and Chemicals

For the extraction of oils from raw materials, CO_2_ cylinders (Food grade and 99.99% pure) were brought from a company (Kosem, Yangsan, Korea). Standard reagents for antioxidant analysis, such as azino-di, 3-ethylbenzthiazoline-6-sulfonic acid (ABTS), 2,2-diphenyl-1-picrylhydrazyl (DPPH), and Trolox, were brought from Sigma-Aldrich (St. Louis, MO, USA). Other chemicals and standards used in this experiment were certified as high-performance chromatography (HPLC) or analytical grade.

### 3.3. Characterization of the Raw Materials

Separated body parts of the JSM were calculated in percentages, and then proximate compositions were analysed. Four major properties, moisture, lipid, protein, and crude minerals were analysed to observe the obtained by-products from the JSM and the quality properties of the specific body portions. An oven was used for analysing the moisture percentages (keeping 105 °C in the oven for 12 h). Soxhlet extraction (*n*-hexane) was considered as total crude oil (Equation (1)). The Kjeldahl method was used for the analysis of protein, and a muffle furnace was used to determine the crude minerals.

### 3.4. Soxhlet Extraction

Soxhlet extraction is a typical method for the extraction of the total amount of oil using *n*-hexane as a solvent [5]. The high solvating power of *n*-hexane can extract both polar and non-polar lipids. In this experiment, we have used 10 g of raw material in a paper thimble with 100 mL *n*-hexane for 12 h. After the whole extraction process, obtained extracts were filtered and the *n*-hexane solvent was separated using a rotary evaporator at 42 °C. This extraction procedure was carried out in triplicate to obtain the result, and the amount of total oil (%) was measured following Equation (1).

(1) $$\mathrm{Total}\;\mathrm{oil}\;(\%)=\;\frac{\mathrm{Weight}\;\mathrm{of}\;\mathrm{oil}}{\mathrm{Weight}\;\mathrm{of}\;\mathrm{sample}}\times 100$$

### 3.5. Supercritical Carbon Dioxide Extraction

Supercritical SC-CO_2_ extraction is a green extraction technique for edible oils from different raw materials. In this experiment, a total of 60 g of freeze-dried raw JSM by-products were taken in a 200 mL extraction vessel according to Figure S1 (Supplementary Material). The inside pressure of the stainless steel-made extractor was increased by a high-pressure pump (Milroyal, Milton Roy, Warminster, PA, USA) that supplied the SC-CO_2_ through the raw materials. Due to the high solvating power and non-polar nature of SC-CO_2_, it can extract the oils from the inner part of the raw materials transfer them to the collector. A back pressure regulator was used to maintain the flow of liquid CO_2_ toward the samples. A suitable temperature (45 °C) and pressure (250 bar). optimized by our previous study [16], were maintained in the whole 3 h extraction process. The extraction was carried out in triplicate to obtain the perfect result. After extraction, the crude oil extract was kept at −24 °C until further analysis. The yield of the oil was calculated following Equation (1), and the percentage of recovery was calculated according to Equation (2).

(2) $$\mathrm{Recovery}\;(\%)=\frac{\mathrm{SC}-{\mathrm{CO}}_{2}-\mathrm{extracted}\;\mathrm{oil}}{\mathrm{Total}\;\mathrm{oil}\;\left(\mathrm{Soxhlet}\;\mathrm{extraction}\right)}\times 100$$

### 3.6. Quality Properties of Oils

#### 3.6.1. Colour and Viscosity

Colour measurements were conducted following our previous study with slight modification [3]. Briefly, we used a portable spectrophotometer, Lovibond RT series, Amesbury, UK. For each operation time, white and black colour standards were used to calibrate the instrument. Viscosity was analysed using a viscometer (Model DVII-Brookfield, Middleboro, MA, USA) with a small S52 adapter. In all the samples, 24 ± 1 °C temperature was maintained using a water bath.

#### 3.6.2. Oxidative Quality of Oils

The oxidative quality of the oils was analysed by evaluating acid value (AV), peroxide value (PV), and free fatty acids value (FFA). AV was expressed as mg KOH/g of oil, PV was determined as meq/kg of oil, and FFA was expressed in percentages. All analyses were conducted using the AOCS official methods, according to the previous study of Franklin et al. [3]. The oxidative quality of the extracted oils was measured in triplicate just after the extraction of oils.

### 3.7. Antioxidant Activities

#### 3.7.1. ABTS^+^ Scavenging Activity

The ABTS^+^ scavenging activity was conducted following our previous work [5]. Briefly, an ABTS solution (7 mM) was prepared in HPLC grade distilled water. Potassium persulfate solution (2.45 mM) was also prepared separately and both solutions were mixed in equal volume in a beaker and kept in a completely dark place for obtaining ABTS^+^ solution. The obtained ABTS^+^ solution was diluted with ethanol (HPLC-grade) until the absorbance of the solution adjusted to 0.70 ± 0.02 at a wavelength of 734 nm. The prepared 3.9 mL ABTS^+^ solution was mixed with 100 µL of the oil sample and kept in a dark place to complete the radical reaction. After completing the reaction, the ABTS^+^ scavenging activity was determined according to the following Equation (3). Trolox was used as standard at a concentration of 100 µg/mL.

(3) $${\mathrm{ABTS}}^{+}\;\mathrm{radical}\;\mathrm{scavenging}\;\mathrm{activity}\;(\%)=[1-(\frac{\mathrm{As}\;-\;\mathrm{Ab}}{\mathrm{Ac}})]\;\times \;100$$

where As indicates the absorbance of the sample, Ac indicates the absorbance of the control (containing only ABTS solution), and Ab indicates the absorbance of the sample mixed with ethanol at 734 nm.

#### 3.7.2. DPPH Radical Scavenging Activity

The DPPH scavenging activity was evaluated following the previous work published from our lab [40] with slight modifications. Briefly, a 0.2 mM DPPH solution was prepared using double distilled water. Afterward, 100 µL of oil was mixed with 3.9 mL DPPH solution and maintained in a dark condition for 30 min to complete the reaction. Reacted solutions were then measured at 517 nm wavelength and the DPPH scavenging activity was analyzed following (Equation (4)). Trolox was used as a standard according to Section 3.7.1. and compared with the obtained results.

(4) $$\mathrm{DPPH}\;\mathrm{radical}\;\mathrm{scavenging}\;\mathrm{activity}\;(\%)=[1-(\frac{\mathrm{As}\;-\;\mathrm{Ab}}{\mathrm{Ac}})]\;\times \;100$$

where, As indicates the absorbance of the sample at 517 nm, Ac is the absorbance of the control (containing only DPPH solution) at 517 nm, and Ab is the absorbance of the oil mixed with ethanol at 517 nm.

### 3.8. Fatty Acid Analysis

The fatty acid composition of the samples was analysed by the following steps. In the first step, oil samples for fatty acid analysis (GC-FID) were prepared according to our previous work [4]. A small amount (0.05 g) of fresh oil was mixed with 0.1% heptadecanoic acid (C_17_H_34_O_2_) mixed with HPLC-grade hexane (2 mL). Afterward, solution was mixed with 3 mL 0.5 N NaOH solution. The mixed solution was mixed thoroughly for about 30 s and heated for nearly 45 min at 75 °C in a sealed bottle. The boiled sample was cooled for 10 min at room temperature and again mixed with 3 mL boron trifluoride (BF3) solution and again subjected to heat at the same temperature (75 °C) for 20 min. After heating, the solution was cooled at room temperature and mixed with 3 m L HPLC-grade hexane and afterward 1 mL 10% NaCl solution. The solution was separated into two parts and the transparent solution from the top parts of the solution was taken using a 3 mL syringe and filtered by a 0.20 µm hydrophobic filter (PTFE-D, Model SD13P020NL, Hyundai micro, Seoul, Korea). The fatty acid composition was analysed by gas chromatography using a 6890 Agilent (Agilent Technologies, Wilmington, NC, USA) gas chromatography following Table S2.

### 3.9. Quality Indexes of Oils

Atherogenic index (AI) and thrombogenic index (TI) are the two important parameters for determining the nutritional quality of the oils, calculated following the method reported by Ulbricht et al. [41]. AI is the indicator of the total saturated fatty acids and the main classes of unsaturated fatty acids following Equation (5). AI is an important indicator of the quality of the oils that can prevent coronary diseases [36,41]. The TI indicates the formation of clots in the blood vessel. According to Ulbricht et al. [41], TI defines the relationships between the prothrombogenic (saturated) fatty acids and antithrombogenic fatty acids (MUFA, ω-6 PUFA, and ω-3 PUFA) (Equation (6)), and the ratio between ω-3 and ω-6 PUFA is considered to be an important quality index of oil [42].

(5) $$\mathrm{AI}=\frac{\left[C12\;:0+\left(4\times C14\;:0\right)+C16\;:0\right]}{\left(\Sigma \mathrm{MUFA}+\;\Sigma \omega -6\;\mathrm{PUFA}+\;\Sigma \omega -3\;\mathrm{PUFA}\right)}$$

(6) $$\mathrm{TI}=\frac{\left[C14\;:0+C16\;:0+C18\;:0\right]}{\left(0.5\times \Sigma \mathrm{MUFA}\right)+\left(\omega -6\;\mathrm{PUFA}\right)+\left(3\times \Sigma \omega -3\mathrm{PUFA}\right)+\left(\frac{\omega -3\;\mathrm{PUFA}}{\omega -6\;\mathrm{PUFA}}\right)}$$

### 3.10. Vitamin-D Analysis

A high-performance lipid chromatography (HPLC) (JASCO HPLC system, Tokyo, Japan) was used to determine the vitamin D (Cholecalciferol (Vitamin-D_3_), Sigma-Aldrich, St. Louis, MO, USA) in the extracted oil samples. Briefly, a 0.5 g of oil sample was mixed with 1 mL chloroform and 1 mL of the previously prepared mobile phase (acetonitrile: methanol, 90:10 (*v*/*v*)). After proper mixing, the oil mixed solvents were filtered through a 0.20 µm hydrophobic syringe filter (Model SD13P020NL, Hyundai micro, Seoul, Korea). A small amount (10 µL) of the filtrate sample was injected into the HPLC system and the analysis of vitamin D was conducted in 265 nm wavelength at 40 °C oven temperature following our previous work [4]. A standard curve of vitamin D, prepared from 1 µg/mL to 1000 µg/mL was also prepared before the analysis of the extracted oils and the chromatograms were shown in Figure S2 (Supplementary Material).

### 3.11. Thermogravimetric Analysis (TGA)

The thermal properties of the extracted oils were analysed in two different parameters as thermogravimetric analysis (TGA) and differential thermogravimetric analysis by following the protocol of our previous work [5,18]. The analysis temperature range was used 50–750 °C and was constantly increased 10 °C/min. A thermogravimetric analyzer was used (Pyris 1 TGA, Perkin Elmer Life and Analytical Sciences, CT, Waltham, MA, USA), whereas nitrogen gas was used for facilitating the suitable reaction condition.

### 3.12. Statistical Analysis

The SPSS software of version 18.00 (Chicago, IL, USA) was applied to analyse the variables. The significant difference of the means was determined by the Duncan Multiple Range tests and the *p* < 0.05 was considered as significant.

## 4. Conclusions

The increasing demand for PUFA-enriched edible fish oil can be mitigated by the proper use of marine fishery resources. Almost 50% of the total body parts of JSM is discarded as by-products, including skin, head, bone, and viscera, which are a rich source of PUFAs. Our findings show that oils extracted from JSM by-products using SC-CO_2_ as an environmentally friendly and cost-effective solvent have huge feasibility for use in the food industry. SC-CO_2_-extracted oils showed better physical, chemical, and biological potentiality compared to the hexane-extracted oils. AI and TI values showed that mackerel oil is safe for food related applications. TGA analysis showed that oils have good thermal stability that can be applied in a wide range of temperatures during food processes. Oils extracted from the by-products are much cheaper than the oils extracted from the fish muscle. Thus, the valorisation of the fish processing wastes meets increasing nutritional demands, and also reduces environmental pollution.

## Figures and Tables

**Table 1:** Colour and viscosity of the SC-CO_2_ and Soxhlet extracted (*n*-hexane) oils.

Colour	SC-CO_2_ Extracted Oils					*n*-Hexane Extracted Oils				
	Skin	Muscle	Bone	Head	Viscera	Skin	Muscle	Bone	Head	Viscera
L*	16.40 ± 0.69 ^f^	25.85 ± 0.79 ^b^	25.56 ± 0.51 ^b^	32.06 ± 0.12 ^a^	18.71 ± 0.32 ^e^	16.35 ± 1.05 ^f^	19.95 ± 0.43 ^d^	21.63 ± 0.58 ^c^	17.00 ± 0.61 ^f^	16.61 ± 0.47 ^f^
a*	3.38 ± 0.42 ^e^	1.12 ± 0.12 ^f^	−2.11 ± 0.13 ^h^	−1.16 ± 0.14 ^g^	3.31 ± 0.15 ^e^	5.23 ± 0.15 ^d^	3.77 ± 0.47 ^e^	9.04 ± 0.15 ^b^	7.78 ± 0.47 ^c^	9.65 ± 0.38 ^a^
b*	19.39 ± 0.42 ^b^	10.22 ± 0.19 ^h^	19.85 ± 0.43 ^b^	11.23 ± 0.23 ^g^	13.25 ± 0.25 ^f^	17.32 ± 0.34 ^d^	18.48 ± 0.28 ^c^	22.36 ± 0.42 ^a^	15.53 ± 0.48 ^e^	17.50 ± 0.47 ^d^
Viscosity										
cP	37.97 ± 0.87 ^g^	20.20 ± 0.46 ^i^	106.40 ± 1.15 ^b^	68.60 ± 0.30 ^d^	8.60 ± 0.10 ^j^	58.70 ± 0.70 ^e^	57.20 ± 0.56 ^f^	145.33 ± 1.20 ^a^	96.13 ± 1.08 ^c^	25.73 ± 0.42 ^h^

±, indicates the standard deviation from the mean; different superscripts indicate the significant differences (*p* < 0.05).

**Table 2:** Oxidative quality indexes of the SC-CO_2_ and Soxhlet extracted (*n*-hexane) oils.

	SC-CO_2_ Extracted Oils					*n*-Hexane Extracted Oils				
	Skin	Muscle	Bone	Head	Viscera	Skin	Muscle	Bone	Head	Viscera
AV (mg KOH/g)	7.76 ± 0.18 ^d^	7.13 ± 0.08 ^f^	7.31 ± 0.18 ^ef^	7.63 ± 0.17 ^de^	7.36 ± 0.15 ^ef^	9.96 ± 0.17 ^c^	10.84 ± 0.25 ^a^	10.73 ± 0.22 ^ab^	10.37 ± 0.25 ^b^	9.66 ± 0.35 ^c^
PV (meq/kg)	1.14 ± 0.12 ^f^	1.26 ± 0.06 ^de^	1.16 ± 0.04 ^ef^	1.28 ± 0.04 ^cd^	1.06 ± 0.04 ^f^	1.61 ± 0.10 ^ab^	1.65 ± 0.06 ^a^	1.53 ± 0.06 ^b^	1.53 ± 0.07 ^b^	1.39 ± 0.04 ^d^
FFA (%)	4.03 ± 0.09 ^d^	3.75 ± 0.09 ^e^	3.41 ± 0.10 ^f^	3.45 ± 0.04 ^fg^	3.29 ± 0.06 ^g^	4.69 ± 0.11 ^a^	4.47 ± 0.09 ^b^	4.20 ± 0.04 ^c^	4.26 ± 0.06 ^c^	4.17 ± 0.05 ^c^

±, indicates the standard deviation from the mean; different superscripts indicate the significant differences (*p* < 0.05).

**Table 3:** Fatty acid compositions (%) of SC-CO_2_ and organic solvent (*n*-hexane) extracted oils.

Name of the Fatty Acids	Supercritical CO_2_ Extracted By-Product Oil					*n*-Hexane Extracted By-Product Oil				
	Skin	Muscle	Bone	Head	Viscera	Skin	Muscle	Bone	Head	Viscera
**Saturated fatty acids (SFA)**										
Myristic	4.58 ± 0.04 ^de^	4.06 ± 0.07 ^g^	4.84 ± 0.08 ^cd^	5.03 ± 0.09 ^bc^	4.38 ± 0.49 ^efg^	5.05 ± 0.07 ^bc^	4.47 ± 0.05 ^ef^	5.33 ± 0.21 ^ab^	4.21 ± 0.09 ^fgh^	3.51 ± 0.08 ^a^
Pentadaecanoic	0.78 ± 0.07 ^de^	0.72 ± 0.02 ^e^	0.89 ± 0.05 ^ab^	0.88 ± 0.03 ^abc^	0.83 ± 0.07 ^bcd^	0.84 ± 0.04 ^bcd^	0.80 ± 0.05 ^def^	0.95 ± 0.05 ^a^	0.77 ± 0.07 ^de^	0.75 ± 0.08 ^de^
Palmitic	23.90 ± 0.22 ^de^	23.73 ± 0.38 ^e^	26.17 ± 0.15 ^b^	26.26 ± 1.30 ^b^	25.87 ± 0.30 ^b^	25.65 ± 0.46 ^bc^	24.76 ± 0.57 ^cd^	27.48 ± 0.41 ^a^	22.49 ± 0.42 ^f^	24.29 ± 0.29 ^de^
Stearic	5.10 ± 0.10 ^d^	5.25 ± 0.14 ^cd^	5.94 ± 0.03 ^c^	5.53 ± 0.27 ^c^	7.54 ± 0.35 ^a^	5.37 ± 0.18 ^cd^	5.28 ± 0.14 ^cd^	5.89 ± 0.35 ^c^	5.18 ± 0.08 ^cd^	7.14 ± 0.15 ^b^
Arachidic	0.64 ± 0.04 ^ab^	0.53 ± 0.02 ^bcd^	0.62 ± 0.03 ^abc^	0.48 ± 0.09 ^cd^	0.51 ± 0.05 ^bcd^	0.68 ± 0.15 ^a^	0.47 ± 0.05 ^d^	0.54 ± 0.09 ^bcd^	0.54 ± 0.05 ^bcd^	0.54 ± 0.04 ^bcd^
Behenic	0.25 ± 0.06 ^a^	0.27 ± 0.03 ^a^	0.28 ± 0.08 ^a^	0.25 ± 0.03 ^a^	0.24 ± 0.03 ^a^	0.24 ± 0.02 ^a^	0.22 ± 0.03 ^a^	0.28 ± 0.07 ^a^	0.22 ± 0.03 ^a^	0.25 ± 0.04 ^a^
Heneicosanoic	0.29 ± 0.01 ^ab^	0.29 ± 0.02 ^ab^	0.27 ± 0.02 ^ab^	0.26 ± 0.05 ^ab^	0.23 ± 0.03 ^b^	0.32 ± 0.05 ^a^	ND	0.29 ± 0.02 ^ab^	0.27 ± 0.03 ^ab^	0.28 ± 0.08 ^ab^
Lignoceric	0.22 ± 0.03 ^ab^	0.22 ± 0.03 ^ab^	0.25 ± 0.05 ^ab^	0.24 ± 0.03 ^bc^	0.24 ± 0.03 ^ab^	0.13 ± 0.02 ^c^	0.18 ± 0.01 ^bc^	0.22 ± 0.03 ^ab^	0.24 ± 0.06 ^ab^	0.26 ± 0.02 ^a^
**Monounsaturated fatty acid (MUFA)**										
Myristoleic	ND	ND	ND	0.12 ± 0.01 ^a^	ND	ND	0.13 ± 0.02 ^a^	ND	ND	ND
Palmitoleic	6.78 ± 0.22 ^c^	5.66 ± 0.10 ^e^	7.43 ± 0.43 ^b^	7.57 ± 0.17 ^b^	5.07 ± 0.16 ^f^	7.22 ± 0.05 ^b^	6.16 ± 0.05 ^d^	7.98 ± 0.25 ^a^	7.36 ± 0.22 ^b^	5.06 ± 0.08 ^f^
Cis-10 heptadecanoic	0.36 ± 0.05 ^c^	0.31 ± 0.10 ^c^	0.32 ± 0.03 ^c^	0.69 ± 0.04 ^a^	0.28 ± 0.06 ^c^	0.33 ± 0.02 ^c^	0.51 ± 0.04 ^b^	0.33 ± 0.07 ^c^	0.27 ± 0.06 ^c^	0.26 ± 0.03 ^c^
Oleic	26.46 ± 0.45 ^f^	26.37 ± 0.44 ^f^	30.51 ± 0.25 ^b^	27.51 ± 0.40 ^e^	24.14 ± 0.14 ^g^	28.61 ± 0.43 ^d^	27.23 ± 0.82 ^e^	32.14 ± 0.27 ^a^	29.43 ± 0.46 ^c^	24.22 ± 0.31 ^g^
Elaidic	0.19 ± 0.05 ^ab^	0.15 ± 0.05 ^b^	0.19 ± 0.06 ^ab^	0.19 ± 0.05 ^ab^	0.22 ± 0.03 ^ab^	0.23 ± 0.05 ^ab^	0.15 ± 0.02 ^b^	0.21 ± 0.04 ^ab^	0.17 ± 0.02 ^ab^	0.24 ± 0.05 ^a^
Eicosenoic	3.26 ± 0.08 ^a^	3.15 ± 0.08 ^ab^	3.02 ± 0.08 ^abc^	2.94 ± 0.04 ^bc^	2.85 ± 0.09 ^c^	3.25 ± 0.04 ^a^	2.97 ± 0.06 ^bc^	3.15 ± 0.26 ^ab^	2.97 ± 0.24 ^bc^	2.89 ± 0.21 ^bc^
Nervonic	0.77 ± 0.02 ^bcd^	0.69 ± 0.05 ^cd^	0.89 ± 0.09 ^ab^	0.75 ± 0.03 ^bcd^	0.81 ± 0.07 ^abc^	0.73 ± 0.03 ^cd^	0.63 ± 0.02 ^d^	0.79 ± 0.15 ^abcd^	0.76 ± 0.13 ^bcd^	0.93 ± 0.07 ^a^
**Polyunsaturated fatty acid (PUFA)**										
Linoleic	1.82 ± 0.06 ^bcd^	1.66 ± 0.09 ^f^	1.88 ± 0.10 ^abc^	2.02 ± 0.08 ^bcd^	3.25 ± 0.07 ^a^	1.93 ± 0.04 ^ab^	1.71 ± 0.03 ^ef^	1.83 ± 0.09 ^b^	1.74 ± 0.20 ^def^	3.01 ± 0.15 ^b^
Linolelaidic	0.16 ± 0.04 ^c^	0.19 ± 0.02 ^bc^	0.21 ± 0.04 ^bc^	0.22 ± 0.03 ^b^	0.17 ± 0.04 ^bc^	0.16 ± 0.02 ^c^	0.28 ± 0.02 ^a^	0.17 ± 0.03 ^bc^	0.17 ± 0.03 ^bc^	0.15 ± 0.04 ^c^
r-Linolenic	0.15 ± 0.03 ^b^	0.37 ± 0.05 ^a^	0.39 ± 0.09 ^a^	0.12 ± 0.02 ^b^	0.39 ± 0.05 ^a^	0.13 ± 0.01 ^b^	0.14 ± 0.01 ^b^	0.42 ± 0.03 ^a^	0.37 ± 0.05 ^a^	0.39 ± 0.06 ^a^
Linolenic	1.08 ± 0.11 ^b^	1.10 ± 0.07 ^b^	0.93 ± 0.02 ^c^	1.10 ± 0.13 ^b^	1.45 ± 0.05 ^a^	0.97 ± 0.07 ^bc^	1.10 ± 0.08 ^b^	0.90 ± 0.02 ^c^	0.93 ± 0.13 ^c^	1.40 ± 0.05 ^a^
Ecosadienoic	1.50 ± 0.04 ^ab^	1.66 ± 0.25 ^a^	1.52 ± 0.11 ^ab^	1.51 ± 0.55 ^ab^	1.30 ± 0.07 ^bc^	1.28 ± 0.17 ^bc^	1.60 ± 0.08 ^ab^	1.34 ± 0.04 ^abc^	1.45 ± 0.37 ^abc^	1.15 ± 0.18 ^c^
Eicosatrienoic	0.19 ± 0.03 ^c^	0.16 ± 0.04 ^c^	ND	2.32 ± 0.02 ^a^	0.29 ± 0.05 ^b^	0.14 ± 0.02 ^c^	0.15 ± 0.03 ^c^	ND	0.15 ± 0.03 ^c^	0.25 ± 0.04 ^b^
Elcosatrienoic	2.81 ± 0.09 ^a^	2.65 ± 0.04 ^ab^	2.26 ± 0.05 ^cd^	2.35 ± 0.15 ^cd^	2.76 ± 0.07 ^a^	2.78 ± 0.16 ^a^	2.70 ± 0.10 ^ab^	2.28 ± 0.08 ^cd^	2.49 ± 0.30 ^bc^	2.19 ± 0.17 ^d^
Arachidonic	0.91 ± 0.04 ^cd^	0.91 ± 0.05 ^cd^	0.79 ± 0.06 ^def^	0.88 ± 0.03 ^de^	1.76 ± 0.07 ^a^	1.05 ± 0.14 ^c^	0.72 ± 0.08 ^ef^	0.65 ± 0.06 ^g^	0.74 ± 0.12 ^def^	1.56 ± 0.16 ^b^
Docosadienoic	0.55 ± 0.08 ^b^	0.51 ± 0.05 ^bcd^	0.41 ± 0.06 ^def^	0.45 ± 0.04 ^bcd^	0.76 ± 0.07 ^a^	0.41 ± 0.08 ^de^	0.52 ± 0.04 ^bc^	0.34 ± 0.05 ^e^	0.35 ± 0.07 ^e^	0.51 ± 0.05 ^bcd^
Eicosapentaenoic	5.81 ± 0.69 ^a^	4.93 ± 0.06 ^a^	3.40 ± 0.06 ^bc^	3.57 ± 0.02 ^bc^	3.81 ± 0.15 ^b^	3.53 ± 0.08 ^bc^	5.14 ± 0.12 ^a^	2.63 ± 0.21 ^c^	2.81 ± 0.29 ^bc^	3.70 ± 0.22 ^b^
Docosahexaenoic	12.56 ± 0.38 ^c^	15.01 ± 0.28 ^a^	6.55 ± 0.19 ^h^	7.34 ± 0.16 ^g^	10.81 ± 0.15 ^d^	8.16 ± 0.04 ^f^	14.51 ± 0.45 ^b^	4.43 ± 0.09 ^i^	6.19 ± 0.17 ^h^	10.27 ± 0.34 ^e^
ΣSFA	35.72	35.07	39.26	38.93	39.84	38.28	36.18	40.98	33.92	37.02
ΣMUFA	37.82	36.33	42.36	33.77	33.37	40.37	37.78	44.60	40.96	33.6
ΣPUFA	27.54	29.15	18.34	21.88	26.75	20.56	28.57	14.99	17.39	24.58
ω- 3/ω- 6	2.97	3.51	1.83	1.25	2.21	2.10	3.55	1.23	1.63	2.43
AI	0.77	0.72	0.87	0.99	0.88	0.87	0.76	0.93	0.77	0.80
TI	0.76	0.75	0.90	0.81	0.90	0.85	0.78	0.92	0.78	0.90

±, indicates the standard deviation from the mean; different superscripts indicate the significant differences (*p* < 0.05).

## Data Availability

Not applicable.

## Supplementary Materials

The following supporting information can be downloaded at: https://www.mdpi.com/article/10.3390/md20010070/s1, Figure S1: A schematic diagram of SC-CO_2_ extraction process; Figure S2: HPLC chromatograms of vitamin D of standards and samples; Table S1: Proximate compositions of JSM raw materials; Table S2: Gas chromatography operating conditions for fatty acid analysis of anchovy oils and commercial oils.
