# Supplementary material for: Lipid Indexes and Quality Evaluation of Omega-3 Rich Oil from the Waste of Japanese Spanish Mackerel Extracted by Supercritical CO2
Source: Mar Drugs. 2022 Jan 13;20(1):70. doi: 10.3390/md20010070 (PMC8780132; doi:10.3390/md20010070)
Supplement: Supplementary file 1 [file marinedrugs-20-00070-s001.zip › marinedrugs-1532797-supplementary.pdf]

Lipid Indexes and Quality Evaluation of Omega-3 Rich Oil from the Waste of Japanese Spanish Mackerel Extracted by Supercritical CO_2

Supplementary File

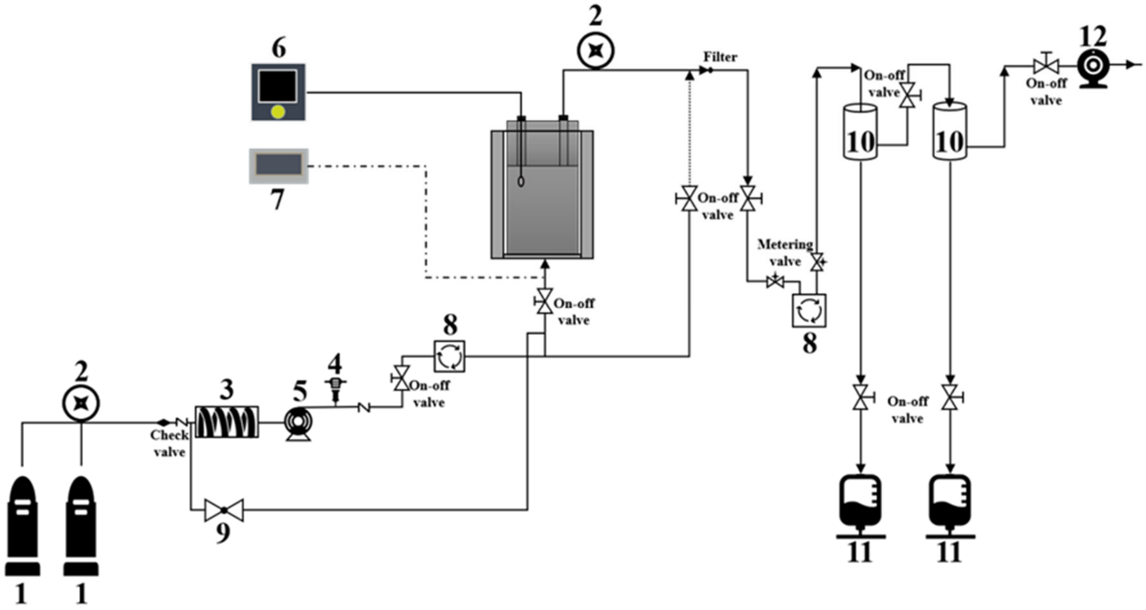

Figure S1. A schematic diagram of SC-CO_2 extraction process.

(1: CO_2 tank, 2: Pressure gauge, 3: Cooling bath, 4: Safety valve, 5: Main pump, 6: Electric indicator, 7: Pressure indicator, 8: Heat exchanger, 9: BPR valve, 10: Separator, 11: Sample collector, 12: Gas flow meter)

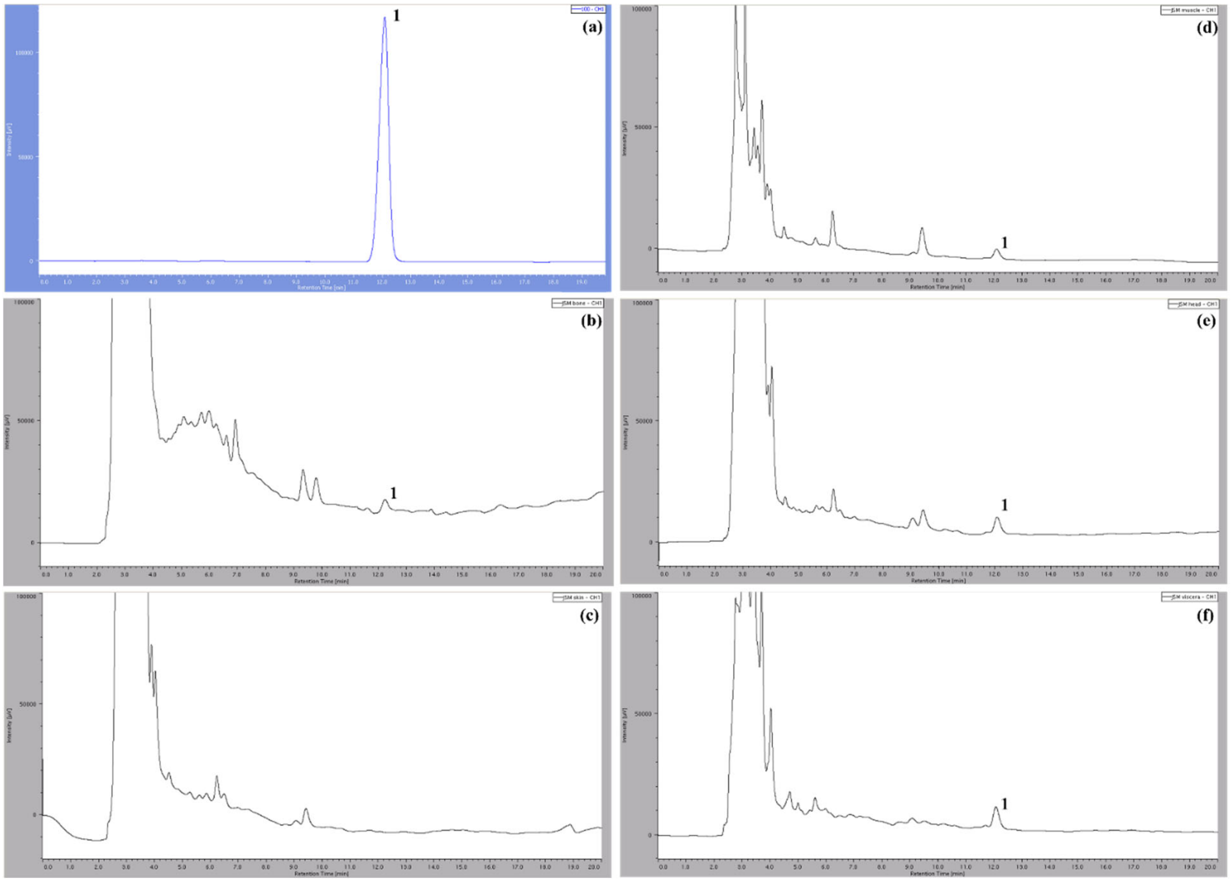

Figure S2. HPLC chromatograms of vitamin D (1) of standards and samples (vitamin D standard (**a**), bone oil (**b**), skin oil (**c**), muscle oil (**d**), head oil (**e**), and viscera oil (**f**))

Table S1. Proximate compositions of JSM raw materials

	Skin	Muscle	Bone	Head	Belly	Others
Body percentage	8.61 ± 0.34[d]	50.89 ± 0.30[a]	7.91 ± 0.14[e]	16.03 ± 0.17[b]	6.22 ± 0.22[f]	9.96 ± 0.13[c]
Moisture	2.28 ± 0.17[d]	11.96 ± 0.51[a]	2.42 ± 0.41[d]	3.87 ± 0.25[c]	11.09 ± 0.73[b]	-
Lipid	51.08 ± 0.01[a]	30.24 ± 0.47[c]	42.50 ± 1.03[b]	42.27 ± 0.96[b]	30.27 ± 1.08[c]	-
Ash	4.78 ± 0.06[d]	6.87 ± 0.37[c]	9.19 ± 0.59[a]	9.09 ± 0.49[b]	4.43 ± 0.74[d]	-
Protein	40.12 ± 0.27[e]	49.14 ± 0.44[b]	45.52 ± 0.34[c]	44.39 ± 0.40[d]	52.17 ± 0.36[a]	-

±, indicates the standard deviation from the mean; different superscripts indicate the significant differences (*P<0.05*)

Table S2. Gas chromatography operating conditions for fatty acid analysis of anchovy oils and commercial oils

Parameters	Conditions
Instrument	Agilent 6890N GC System
Split	Splitless
Inject Temperature	250 °C
Carrier Gas & Flow Rate	He, 0.5 mL/min
Oven Condition	140 °C (5 min) → 4 °C/min for 25 min → 240°C (17 min)
Detect Temperature	260 °C
Column	Supelco sp. 2560, capillary column<br>Fused silica Capillary 100.0 m × 250 μm × 0.2 μm film thickness
